# Correlation between clinical classification and genetic analysis of familial hypercholesterolemia in premature coronary artery disease in a cohort of Egyptian patients

**DOI:** 10.1186/s40246-025-00769-y

**Published:** 2025-06-14

**Authors:** Rania A. Zahwo, Ziad N. Rezk, Tamer M. Elwasify, Amr M. Zaki, Hoda M. El Assi, Eman Ramadan, Abdallah Y. Habib, Wael A. Hassan, Ahmed Abdel-Raouf, Ameera Ragheb, Amin F. Shaker, Khaled E. Amer, Heba Sh. Kassem

**Affiliations:** 1 Clinical Genomics Center, Faculty of Medicine, Alexandria University, Alexandria, Egypt; 2 Medical Genetics Department, Armed Forces College of Medicine, Cairo, Egypt; 3 Cardiology Department, Armed Forces College of Medicine, Cairo, Egypt; 4 Cardiology Department, Faculty of Medicine, Alexandria University, Alexandria, Egypt; 5 Pharmacology and Toxicology Department, Faculty of Pharmacy, The British University in Egypt, Cairo, Egypt; 6 Clinical Pathology Department, Military Medical Academy, Cairo, Egypt; 7 Egypt Center for Research and Regenerative Medicine, Cairo, Egypt

**Keywords:** DLCN, Familial hypercholesterolemia, Genetic testing, Premature CAD, IHD, Egypt

## Abstract

**Background:**

Familial Hypercholesterolemia (FH) is a major risk factor for premature Coronary Artery Disease (CAD). Genetic testing is the gold standard for FH diagnosis. The purpose of this Observational Analytical Cross-sectional study was to estimate the proportion of genetically confirmed Familial Hypercholesterolemia in Patients with premature Coronary Artery Disease in a cohort of Egyptian patients.

**Methods:**

Next generation sequencing (NGS) was conducted for 7 genes (***LDLR****, ****PCSK9****, ****APOB****, ****APOE****, ****ABCG5****, ****ABCG8*** and ***LDLRAP1***) commonly associated with FH in 94 patients with Premature CAD from 2 tertiary hospitals in Cairo and Alexandria, Egypt. Individuals were clinically assessed using the Dutch Lipid Network criteria and genetically-confirmed FH prevalence was analyzed.

**Results:**

Fourteen patients had pathogenic or likely pathogenic mutations in ***LDLR****, ****APOB****, ****PCSK9*** and ***LDLRAP1*** genes. Three patients had homozygous autosomal dominant FH and another 3 patients had autosomal recessive hypercholesterolemia. In addition, 10 patients had rare variants of uncertain significance in ***LDLR****, ****APOB****, ****APOE****, ****ABCG5*** and ***ABCG8*** genes*.*

**Conclusions:**

The prevalence of genetically confirmed FH in premature CAD (PCAD) patients in this study was found to be 14.89%. The Dutch Lipid Clinic Network (DLCN) scoring system is suggested as a good screening tool for familial hypercholesterolemia but confirmatory genetic testing is essential for the accurate diagnosis and management of the patients. In Egypt, the high rate of consanguinity contributes to the high prevalence of both homozygous autosomal dominant and recessive FH.

**Supplementary Information:**

The online version contains supplementary material available at 10.1186/s40246-025-00769-y.

## Background

Coronary Artery Disease (CAD) is a progressive medical condition caused by insufficient vascularization of the myocardium due to accumulation of plaques [1]. It ranks first in the global causes of death; it was responsible for approximately 16% of total deaths in 2019 [2]. Early-onset CAD is defined as the onset of events prior to the age of 40 years. Premature Coronary artery disease (PCAD) is the occurrence of symptomatic CAD prior to the ages of 55 years and 65 years in males and females, respectively [3].

Familial hypercholesterolemia (FH) is defined as an elevation of plasma low-density lipoprotein levels linked to a genetic cause; this elevation leads to the early onset of severe atherosclerosis and subsequent early onset of CAD [4].

The global prevalence of familial hypercholesterolemia ranges from 1 in 200 to 1 in 500 [5]. The prevalence of FH in CAD patients younger than 45 years is approximately 1 in 7 individuals [6]. Patients with untreated FH have a 13 fold higher risk of premature IHD compared to the general population [7]. In addition, the risk of coronary artery disease (CAD) is increased six-fold in those with high levels of LDL-C 190 mg/dL and 22-fold at the same LDL-C level when a genetic variant causing FH is present [8].

Three different sets of clinical criteria are commonly used to predict FH; the Simon Broome Diagnostic Criteria (SBR), developed and commonly used in the United Kingdom, the Dutch Lipid Clinic Network criteria (DLCN) and the Make Early Diagnosis to Prevent Early Death criteria (MEDPED). Genetic testing remains the gold standard for diagnosing FH [5]^.^

Around 20% of patients with premature CAD have a FH causative mutation explaining the earlier onset of their condition [9]. In Egypt, the estimated prevalence of FH in premature CAD patients based on the Dutch Lipid Clinic Network criteria is around 17% [10]. These findings were based on clinical criteria only and no genetic testing was performed, making this study an early step towards identifying the prevalence of genetically confirmed FH in premature CAD patients in Egypt.

Cascade screening is of utmost importance in FH management as it is estimated that fewer than 20% of individuals with familial hypercholesterolemia globally have been diagnosed, and less than 10% receive sufficient management [5].

In Egypt, Reporting of FH mutations is lacking, and higher reporting rates would lead to early preventative measures for premature CAD, cascade screening of susceptible families, and the application of an appropriate lipid-lowering regimen. These measures would result in lowered morbidity and mortality associated with the complications of FH [11].

This study is based on the hypothesis that individuals with premature CAD would have a higher prevalence of mutations in common Familial Hypercholesterolemia genes.

In this study, genetic testing was conducted to identify the frequency of mutations in the most common causative genes of hereditary single gene familial hypercholesterolemia. It was also aimed to be a step toward unraveling the mutations in the Egyptian population as a pilot study to aid in the future application of a personalized approach to the prevention, diagnosis, and management of FH. These genes are ***LDLR*** (Low-density lipoprotein receptor), ***APOB*** (Apolipoprotein B), ***PCSK9*** (Proprotein convertase subtilisin/Kexin type 9), and ***APOE*** (Apolipoprotein E), which are responsible for autosomal dominant familial hypercholesterolemia. ***LDLRAP1*** (Low-density lipoprotein receptor adaptor protein 1) which is responsible for autosomal recessive hypercholesterolemia. In addition to ***ABCG5/8*** (Adenosine triphosphate-binding cassette subfamily G members 5 and 8) genes causing sitosterolemia, which is considered FH phenocopy and may mimic FH [12, 13].

## Methods

The study was conducted on 96 premature CAD patients. They were recruited from 2 tertiary hospitals in Cairo and Alexandria, Egypt.

All analyses carried out through this study were pre-planned, with the primary goal of confirming the hypothesis that individuals with premature CAD would have a higher prevalence of mutations in common Familial Hypercholestrolemia genes.

### Study design and setting

This study is an Observational Analytical Cross-sectional study. Patients with premature CAD were selected for the study from patients at Kobri El Qobba Cardiology hospital’s wards, critical care units and emergency unit in Cairo and Smouha University hospital, Cath lab unit in Alexandria, in the period from June 2022 to May 2023. All patients were diagnosed with CAD through angiography. An informed consent for participation in the study was taken for each patient. Those patients under the age of 18 years of age were included following an informed consent from the parents or surrogate after taking the assent of the child. A full clinical evaluation and detailed family history with pedigree construction was undertaken for every patient. DLCN scores were calculated during the same visit. Also, a full lipid profile was performed for most patients. NGS (Next Generation sequencing) was performed at the Egypt Centre for Research and Regenerative Medicine based in Cairo.

### Study population and sampling

Inclusion criteria were males < 55 and females < 60 years of age with premature CAD. Patients under 40 years of age with CAD are designated as early-onset CAD and are also included in the study. Diagnosis of CAD is established through finding a significant lesion on a coronary angiogram. Those excluded from the study were those with thyroid dysfunction, hepatic or renal disease. Diabetics and patients over 40 years of age with a negative family history of PCAD were excluded from the Alexandria cohort.

Sampling was done through non-probability convenience sampling, where any patient fitting the inclusion criteria attending Kobri El Qobba or Smouha hospitals was offered participation in the study.

The current study’s sample size was calculated using Epi INFO version 7.2.4.0 (Centers for Disease Control and Prevention (CDC), Atlanta, Georgia) based on a study, which reported the prevalence of genetically confirmed Familial Hypercholesterolemia in patients with PCAD to be 8.7% [14]. The precision limit could be increased up to 10% but cannot surpass the disease prevalence. Based on these data, for a sample size needed at a confidence level of 95% and precision (margin of error) of 8.0%, 48 subjects for each cohort was considered satisfactory.

### Clinical and laboratory evaluation

Patients with confirmed premature CAD were contacted, the research was explained to them, and those who agreed to participate were scheduled an interview. A total of 96 patients participated in the study, 48 patients from each hospital were subsequently interviewed. During the interview, procedures of participation were explained to patients, a written informed consent was taken from each patient, a clinical sheet was used for collecting relevant history, two peripheral venous blood samples were collected per patient; one for a full lipid profile and another in Ethylenediaminetetraacetic acid (EDTA) collection tubes for DNA extraction.

Clinical data were collected from the patients, including age at disease onset, consanguinity, weight and height used for BMI calculation, presence of CAD risk factors such as family history of premature CAD or FH, smoking, hypertension and diabetes, lipid lowering drugs intake and the CAD diagnostic method used. DLCN score was calculated for each patient as a scoring system for FH [15].

Levels of LDL, to be used for DLCN scoring, were calculated according to the Friedewald formula (16). Most of our patients were already on statins with no knowledge of their baseline lipid profile. This led to the absence of a baseline LDL level for all patients. However, it was estimated according to the type and dose of the lipid-lowering treatment before applying the DLCN criteria. Estimated on-treatment LDL-C levels for CAD patients of over 55 mg/dl, which is the target on-treatment LDL levels for CAD patients, were used to determine the presence of hypercholesterolemia [17, 18].

### Genetic testing and variant interpretation

Genomic DNA was extracted using Chemagic DNA blood 400 kit h96 (Revvity, Baesweiler, Germany) according to the manufacturer’s protocol. DNA concentration and quality were assessed using Nanodrop and Qubit Flurometer (Thermo Fisher Scientific, Waltham, Massachussets). Qubit dsDNA BR Assay kit was used in this step (Thermo Fisher Scientific, Waltham, Massachussets).

Microfluidic electrophoretic separation of nucleic acids was conducted on LabChip GXII Touch using the Genomic DNA reagent kit (Revvity, Baesweiler, Germany). Library preparation was performed using the DNA prep with enrichment kit (Illumina, San Diego, California), followed by DNA quantitation with Qubit dsDNA HS assay kit (Thermo Fisher Scientific, Waltham, Massachussets). Library quality was assessed using DNA NGS 3 K Reagent kit (Revvity, Baesweiler, Germany) on the LabChip GXII Touch platform. Quality was assessed following DNA fragmentation and after library preparation.

DNA sequencing was performed on the Illumina NextSeq 2000 sequencer using a 2 × 151 bp protocol on a P1 300 flow cell. TruSight Cardio Sequencing kit (Illumina, San Diego, California), which uses Streptavidin Magnetic Beads to capture probes hybridized to targeted regions at only the exons and flanking sequence of 3–35 base pairs of 174 genes related to different inherited cardiac conditions. It was used as it includes the 7 most commonly reported genes related to FH (*LDLR, APOB, APOE, PCSK9, ABCG5, ABCG8, LDLRAP1)*. Possible deletions or variants in promoter regions or introns were not evaluated.

Alignment and variant calling were performed on NextSeq 2000’s onboard DRAGEN secondary analysis system. High quality reads (Q > 33 reads/base) were mapped to hg19 (Human Genome 19 reference genome). Samples had a minimum read depth of 52.16 × and an average depth of 129.42x. Annotation and variant analysis were done using Varsome v11.15 (https://varsome.com/) and Franklin v.73 (https://franklin.genoox.com/clinical-db/home) platforms. The American College of Medical Genetics (ACMG) guidelines were applied in variant scoring and each variant was also assessed based on pathogenicity scores, effect on target protein and conservation scores [19]. ***LDLR*** variant analysis was done according to the ClinGen Familial Hypercholesterolemia Expert Panel Specifications to the ACMG/AMP Variant Classification Guidelines version [20]. Analysis included the coding exons of the aforementioned 7 genes, in addition to the flanking sequence of 3–35 base pairs.

### Statistical analysis

Data were entered on Microsoft Office Excel Program for Windows, 2013. All statistical analyses were performed using SPSS (Statistical Package for Social Sciences) version 23 (IBM SPSS Statistics, IBM Corporation, Armonk, New York).

Normality of data was assessed using Kolmogorov–Smirnov statistical normality test. Quantitative data are presented as mean ± standard deviation (SD) or median (interquartile range [IQR]), unless otherwise specified. Qualitative data are presented as frequency or percentage.

The Chi-square test was used to compare frequencies and differences, while quantitative variables were evaluated using the Student T test or Mann–Whitney U test. *P* values ≤ 0.05 were considered statistically significant.

## Results

### Clinical and demographic characteristics of the study cohort

The purpose of this study was to investigate the prevalence of genetically confirmed FH in premature CAD patients in 2 tertiary hospitals in Cairo and Alexandria. The recruited patients were 96 patients, 48 patients from each hospital. The age of CAD onset in the test sample ranged from 14 to 55 years and the median age of onset was 44 (IQR 11) years. Positive family history of premature CAD was detected in 57 (59.37%) patients and 39 (40.62%) had a positive family history of dyslipidemia.

The patients’ risk factors, including hypertension, diabetes mellitus, smoking and obesity were sought for among the participants and it was found that 63 (65.62%) of the cohort were smokers, 42 (43.75%) were hypertensive and 18 (18.75%) were diabetic.

BMI was calculated for each patient as a measure of obesity; the mean BMI was 28.83 ± 4.96. Only 21 patients (21.87%) were in the healthy weight range (< 25), 36 patients (37.5%) were in the overweight range (25–29.9), 30 (31.25%) were in the class 1 obesity range (30–34.9), 7 (7.29%) were in the class 2 obesity range (35–39.9) and 2 patients (2.08%) were in the class 3 obesity range (> 40).

The median LDL level of the studied cohort was 116 (94.1) mg/dl, ranging from 29 to 655.5 mg/dl. Of the 96 patients, 82 (85.41%) were on lipid lowering agents.

It is worth mentioning that only 6 patients (6.25%) showed controlled on-treatment LDL levels (Target LDL < 55 mg/dl) and all 6 patients were on high intensity statins. Table 1 shows the clinical and demographic data of both study cohorts and the comparison between the Cairo and Alexandria cohorts. Two patients had no data for their lipid profile. These missing data were excluded from analysis and statistical analysis regarding lipid profiles was performed for the remaining 94 cases.

**Table 1:** Test sample characteristics (N = 96)

	Both cohorts N = 96	Alexandria cohort N = 48 (%)	Cairo cohort N = 48	*P* value	Missing values (N)
Males n (%)	83 (86.45)	35 (72.9)	48 (100)	< 0.0001	–
Age of CAD onset, years, n (%)				< 0.0001	–
< 40	33 (34.37)	26 (54.16)	7 (14.58)		
40–49	45 (46.68)	20 (41.66)	25 (52.08)		
50–55	18 (18.75)	2 (4.16)	16 (33.33)		
Age of CAD onset, years—median (IQR)	44 (11)	39 (8)	48 (7)	U = 28.364 *P* < 0.001	–
Body mass index—mean ± SD	28.83 ± 4.96	27.6 ± 5.0	30.06 ± 4.70	t = −2.482 *P* = 0.015	–
Hypertensive—n (%)	42 (43.75)	19 (39.58)	23 (47.91)	0.411	–
Diabetic—n (%)	18 (18.75)	0 (0)	18 (37.5)	< 0.0001	–
Smoker—n (%)	63 (65.62)	28 (58.33)	35 (72.91)	0.133	–
Family history of premature CAD—n (%)	57 (59.37)	38 (79.17)	19 (39.58)	< 0.0001	–
Family history of dyslipidemia—n (%)	39 (40.62)	17 (35.41)	22 (45.83)	0.299	–
Positive consanguinity—n (%)	32 (33.33)	19 (39.58)	13 (27)	0.24	–
Cholesterol, mg/dl—median (IQR)	185 (93)	220 (96)	169.50 (55)	U = 13.5 *P* = 0.001	2
LDL, mg/dl—median (IQR)	116 (94.1)	156.5 (98.5)	97.5 (49.95)	U = 13.5 *P* = 0.001	2
HDL, mg/dl—median (IQR)	36.8 (13)	38 (15)	43.50 (12)	U = 6 *P* = 0.025	2
Triglycerides, mg/dl—median (IQR)	144.5 (116)	141 (89)	152 (120.75)	U = 0.167 *P* = 0.838	2
On lipid lowering agent—n (%)	82 (85.41)	37 (77.1)	45 (93.75)	0.021	–
DLCN (Probable or definite)-n (%)	38 (39.58)	23 (47.91)	15 (31.25)	0.16	–

Values are presented as mean ± SD, n (%), or median (IQR)

T Student T test, U = Mann–Whitney U test

*CAD* Coronary Artery Disease, *IQR* Interquartile Range, *LDL* Low-density lipoprotein, *HDL* High-density lipoprotein, *DLCN* Dutch Lipid Clinic Network criteria

Clinical evaluation for FH was conducted based on the DLCN criteria, this revealed 15 (15.62%) patients with unlikely FH (Score < 3), 43 (44.79%) patients with possible FH (Score 3–5), 25 (26.04%) patients with probable FH (Score 6–8) and 13 (13.54%) patients with definite FH (Score more than 8).

In the Cairo cohort, 100% of the cases were males, whereas males represented 72.9% of the cases in the Alexandria cohort. Additionally, the median age of CAD diagnosis was 39 years in Alexandria and 48 years in Cairo, with a statistically significant difference between the 2 groups.

The patients'risk factors revealed statistically significant differences between the two groups in terms of mean BMI, diabetes prevalence, and family history of premature CAD. The Alexandria cohort had a lower mean BMI (27.6 ± 5.0) compared to the Cairo cohort (30.06 ± 4.70). None of the patients in the Alexandria cohort were diabetic, whereas 18 patients (18.75%) of the total cohort had diabetes. Additionally, approximately 80% of the cases in the Alexandria cohort had a positive family history of premature CAD, which is twice the proportion found in the Cairo cohort. There were no statistically significant differences between the two groups in terms of hypertension, smoking, or family history of dyslipidemia.

Regarding the lipid profile, patients in the Alexandria cohort had significantly higher median LDL-C levels [156.5 mg/dL (IQR: 98.5)] and total cholesterol levels [220 mg/dL (IQR: 96)] compared to the Cairo cohort, which had median LDL-C levels of 97.5 mg/dL (IQR: 49.95) and total cholesterol levels of 169.5 mg/dL (IQR: 55). These differences were statistically significant between the two groups. However, there was no statistically significant difference between 2 groups in the number of patients clinically diagnosed with definite or probable FH according to the DLCN criteria.

Out of the 96 samples collected, 94 samples were sequenced successfully and two samples failed quality checks and were excluded from downstream analysis. Among the 94 cases sequenced 14 (14.89%) patients had pathogenic and likely pathogenic variants, 10 (10.64%) patients have rare variants of uncertain significance (VUS), and 70 (74.47%) patients have negative/common VUS/carriers of *ABCG5/8,* results as shown in Table 2.

**Table 2:** Genetic testing results

Genetic testing	Total study cohort (n = 94)	
	N	%
Pathogenic/likely pathogenic	14	14.89
VUS	10	10.64
Negative/common variants	70	74.47

*VUS* Variant of Uncertain Significance

Eight pathogenic variants in the ***LDLR*** gene were found in 9 patients. Among these 9 patients; 3 were homozygous, one was compound heterozygous, while the remaining 5 were heterozygous. Two pathogenic ***LDLRAP1*** gene variants were detected in 3 cases. In addition, one patient had a pathogenic variant in the ***PCSK9*** gene and one patient had a likely pathogenic variant in the ***APOB*** gene. The pathogenic and likely pathogenic variants detected in the studied cases are presented in Table 3

**Table 3:** pathogenic/likely pathogenic variants

Variant ID	Variant type	ACMG classification	Affected patients in current study in relation to DLCN
*LDLR*			
NM_000527.5:c.1255 T > G (p.Tyr419 Asp)	SNV missense	Pathogenic PS1,PM2,PP1_moderate,PP3, PP4,PS4_supporting	Homozygous variant in 1 patient with definite FH
NM_000527.5:c.1567G > A (p.Val523Met)	SNV missense	Pathogenic PS1,PS4, PM5_strong,PM2,PP3	Heterozygous variant in 1 patient with possible FH and a compound heterozygous variant in 1 patient with definite FH
NM_000527.5:c.1414G > T (p.Asp472 Tyr)	SNV missense	Likely pathogenic PS4,PM2	
NM_000527.5:c.1846-1G > A	SNV Splice acceptor	Pathogenic PVS1, PM2, PS4, PP3, PP4	Heterozygous variant in 1 patient with probable FH
rs1057519667 NM_000527.5:c.1187 del G	Deletion frameshift	Pathogenic PVS1, PM2, PS4, PP4	Heterozygous variant in 1 patient with probable FH
NM_000527.5:c.1999 T > C (p.Cys667 Arg)	SNV missense	Pathogenic PS4, PS3, PP3, PM2, PS3,PP4	Homozygous variant in 1 patient with definite FH
NM_000527.5:c.2416 dup (p.Val806fs)	Insertion Frameshift	Pathogenic PVS1, PM2, PS4, PP4	Homozygous variant in 1 definite FH patient and heterozygous variant in 1 patient with probable FH
NM_000527.5:c.1195G > A (p.Ala399 Thr)	SNVMissense	Likely pathogenic PS4, PM2, PP3, PP4	Heterozygous variant in 1 patient with definite FH
*PCSK9*			
NM_174936.4:c.644G > A (p.Arg215His)	SNV missense	Pathogenic PS4, PM2, PM5, PM1	Heterozygous variant in 1 patient with probable FH
*APOB*			
NM_000384.3:c.13477 CAG (p.Gln4494 del	Inframe deletion	Conflicting classifications of pathogenicity Likely pathogenic(4); VUS(4); Likely benign(2) (Clinvar) According to ACMG evaluation in current study: Likely pathogenic PS3, PM2, PM4, PP3	Heterozygous variant in 1 patient with possible FH
*LDLRAP1*			
NM_015627.3:c.604 del (p.Ser202fs)	Deletion Frameshift	Pathogenic PVS1, PM2, PP5	Homozygous variant in 2 patients, one with possible FH and the other with definite FH
NM_015627.3:c.603 dup (p.Ser202fs)	Insertion Frameshift	Pathogenic PVS1, PM3, PP5	Homozygous variant in 1 patient with definite FH

*LDLR* Low-density lipoprotein receptor, *PCSK9* = proprotein convertase subtilisin/kexin type 9, *ApoB* Apolipoprotein B, *LDLRAP1* Low-density lipoprotein adaptor protein 1, *SNV* single nucleotide variant, *FH* Familial hypercholesterolemia, *PVS* pathogenic very strong, *PS* pathogenic strong, *PM* pathogenic moderate, *PP* pathogenic supporting

Ten patients had rare variants of uncertain significance (VUS) in the ***LDLR****, ****APOB****, ****APOE****,* and ***ABCG5/8*** genes, with no rare VUS detected in the ***PCSK9 and LDLRAP1*** gene. Specifically, two patients had VUS in ***LDLR*** gene, 5 in ***APOB*** gene, 1 in ***APOE*** gene, 1 compound heterozygous variants in ***ABCG5*** and 1 compound heterozygous variants in ***ABCG8*** (Supplemental Table I). Common VUS and heterozygous variants in ABCG5/8 are listed in Supplemental Tables II and III.

Two APOE variants were found in 16 cases, (APOE):c.388 T > C (p.Cys130 Arg) was detected in 14 patients, and (APOE):c.487 C > T (p.Arg163 Cys) was detected in 2 cases. In addition, a drug response variant (APOE):c.526 C > T (p.Arg176 Cys) was detected in 10 patients in which patients with CT or TT alleles were reported to have better response to atorvastatin than patients with CC allele.

Table 4 shows detected novel and hot VUS, which are the most suspicious variants and can be upgraded to pathogenic/likely pathogenic by obtaining additional proof of pathogenicity through functional studies or segregation analysis [21].

**Table 4:** Novel and hot Variants of uncertain significance (VUS) detected in the present study

Variant ID	Variant type	Clinvar and ACMG classification	GnomAD Allele frequency (version 4.1.0)	exon number/relation to functional domain	Insilico prediction
*LDLR*					
NM_000527.5:c.431 C > T (p.Pro144Leu)	SNV missense	VUS PM1,PM2,PS4_supporting	0.000003718	Exon 4/LDL-receptor class A3	REVEL: benign supporting 0.392 SIFT: uncertain Mutation Taster: benign supporting
NM_000527.5:c.1546G > A (p.Gly516Ser)	SNV missense	Conflicting classifications of pathogenicity VUS(8); Likely benign(3); Benign(1) PM2, PS4_supporting	0.00007125	Exon10/LDL-receptor class B 3	REVEL: benign moderate 0.347 MutationTaster: uncertain SIFT: uncertain
*APOB*					
NM_000384.3:c.9386 C > G (p.Pro3129 Arg)	SNV missense	VUS PM2, PP3	Not found	Exon 26	REVEL: benign Supporting0.456 SIFT: pathogenic VariantTaster: benign
NM_000384.3:c.2062 A > T (p.Ile688Phe)	SNV missense	VUS PM2, BP4	Not found	Exon 14	REVEL: Benign Strong0.046 SIFT: benign moderate VariantTaster: benign supporting
ABCG5*					
NM_022436.3:c.1285G > A (p.Ala429 Thr)	SNV missense	VUS PM2, PP3	0.0000397	Exon 9/ABC transmembrane type-2	REVEL: Benign Supporting 0.383 Polyphen possibly damaging SIFT: Deleterious
NM_022436.3:c.1426G > C (p.Val476Leu)	SNV missense	VUS PM2	Not found	Exon 10/ABC transmembrane type-2	REVEL Benign Strong0.087 SIFT: benign VariantTaster: uncertain

*LDLR* Low-density lipoprotein receptor, *ApoB* Apolipoprotein B, *ABCG5/8* ATP binding cassette subfamily G member 5/8, *SNV* single nucleotide variant, *VUS* variant of uncertain significance, *FH* Familial hypercholesterolemia, *BP* benign supporting, *BS* benign strong, *PM* pathogenic moderate, *PP* pathogenic supporting, ABCG5* compound heterozygous variant

### Clinical and genetic data correlation

**Fig. 1:**
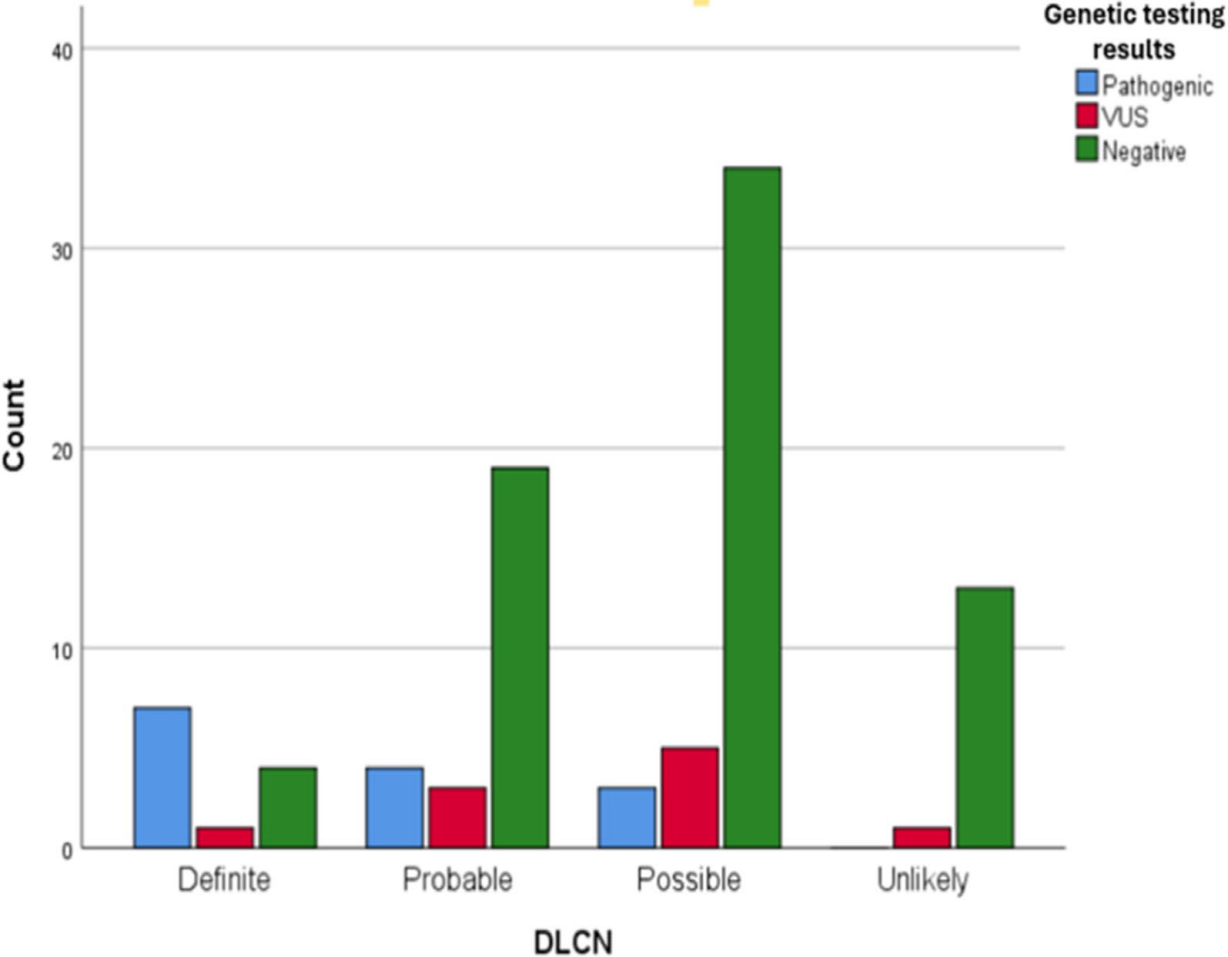
Genetic testing results across different DLCN classifications of FH

When comparing DLCN with genetic findings, it can be seen that 7 patients with definite FH have pathogenic or likely pathogenic variants, 1 patients have rare VUS, and 4 patients have negative results/common variants/ABCG5/8 carrier. Among the 26 cases with probable FH, 4 patients have pathogenic/likely pathogenic variants, 3 patients have VUS and 19 patients have negative results/common variants/ABCG5/8 carriers. The majority of patients (76%) with possible FH (34 cases) have negative results/common variants/ABCG5/8 carriers, whereas 5 patients have rare VUS and only 3 patients have pathogenic variants. In contrast, none of the patients unlikely to have FH have pathogenic variants, 1 patients has rare VUS, and 13 patients have negative/common variants/ABCG549./8 carriers as shown in Table 5 and Fig. 1. In addition, 2 cases have no results due to failed sequencing.

**Table 5:** Genetic testing results across different DLCN classifications of FH

N = 94	Pathogenic/likely pathogenic n (%)	Rare VUS n (%)	Negative/common variants/*ABCG5/8* carriers n (%)
Definite FH (Score more than 8)	7/12 (58.33)	1/12 (8.33)	4/12 (33.33)
Probable FH (Score 6–8)	4/26 (15.38)	3/26 (11.54)	19/26 (73.08)
Possible FH (Score 3–5)	3/42 (7.14)	5/42 (11.9)	34/42 (80.95)
Unlikely FH (Score less than 3)	–	1/14 (7.14)	13/14 (92.86)

*FH* familial hypercholesterolemia, *VUS* variant of uncertain significance

Table 6 assesses the predictive values for the presence of pathogenic or likely pathogenic variants in patients diagnosed by DLCN criteria as definite or probable FH versus patients with possible or unlikely FH after the exclusion of patients with rare VUS Characteristics of patients with and without FH-related variants were compared in Table 7. The patients were classified into 3 groups: patients with pathogenic variants, patients with VUS and patients with negative results. Age at diagnosis, total cholesterol, LDL, HDL and triglycerides were compared among the groups. Statistically significant differences were found in respect to age of CAD onset, LDL and total cholesterol levels between patients harboring pathogenic variants and those with VUS or negative results. However, there were no statistically significant differences in age of CAD onset or lipid profiles between patients with common VUS and those with negative results.

**Table 6:** The predictive values of DLCN criteria versus genetic diagnosis

Clinical diagnosis	Pathogenic/likely pathogenic	Negative	Sensitivity (%)	Specificity (%)	PPV (%)	NPV (%)
Definite/probable	11 (TP)	23 (FP)	78.6%	67.14%	32.3%	94%
Possible/unlikely FH	3 (FN)	47 (TN)				

*TP* true positive, *FP* false positive, *FN* false negative, *TN* true negative, *PPV* Positive Predicted Value, *NPV* Negative Predicted Value, *FH* Familial Hypercholesterolemia

PPV: TP/TP + FP; NPV: TN/TN + FN; Sensitivity: TP/TP + FN; specificity: TN/TN + FP

**Table 7:** Characteristics of patients with and without FH variants

		Pathogenic/likely pathogenic N = 14	Rare VUS N = 10	Common VUS/carriers of *ABCG5/8* variants/Negative N = 70	Missing data	*P* value
Age of CAD onset		27.93.82	48	51.34	–	0.013
LDL		73.93	51.55	41.64	2	> 0.001
HDL		58.64	40.75	46.24	2	0.212
Triglycerides		36.36	49.25	49.48	2	0.253
Cholesterol		73.29	54.45	41.35	2	> 0.001

Data is presented as mean rank

*LDL* Low-density Lipoproteins, *HDL* High-density Lipoproteins, *FH* Familial Hypercholesterolemia

Statistical analysis was performed using the Kruskal–Wallis test

## Discussion

This study aimed to investigate the prevalence of causative variants of familial hypercholesterolemia in patients with early and premature CAD (Age < 55 in males and < 65 in females). The cumulative exposure to high LDL-C levels in FH patients results in an elevated LDL-C burden (LCB), which is calculated by multiplying the initial LDL-C levels by patients’ age at diagnosis and adding annual LDL-C levels in follow-up visits, increasing the risk of atherosclerotic coronary artery disease. In addition, high LCB can modulate the innate immunity, which plays a role in the pathogenesis of atherosclerosis; however, both factors can act independently [22].

DNA was extracted from 96 blood samples collected from patients attending Tertiary cardiovascular centers for treatment of CAD in Cairo’s Kobri ElKoba hospital and Alexandria’s Main University hospitals and analyzed for variants in coding sequence of ***LDLR****, ****PCSK9****, ****APOB****, ****APOE****, ****LDLRAP1****, ****ABCG5*** and ***ABCG8*** using NGS.

Among the 94 successfully sequenced cases, 14 (14.89%) patients have pathogenic/likely pathogenic variants. By comparing the demographic data of the 2 cohorts (Table 2), statistically significant differences are seen regarding age of disease onset, diabetes presence and positive family history of PCAD. These differences are explained by the difference in exclusion criteria between the 2 cohorts, as patients over 40 years of age with a negative family history of PCAD and all diabetics were excluded from the Alexandria cohort while they were included in the Cairo cohort. However, there was no statistically significant difference in the frequency of patients with definite/probable FH according to DLCN between the 2 cohorts.

Diabetes Mellitus is widely considered as an independent risk factor for CAD. Studies have shown that people with type 2 DM have a 2 to 3 times higher chance of developing CAD than the general population and that risk is further doubled in case of patients with both FH and DM [23, 24]. DM is also implicated in the development of dyslipidemia. Although, it has also been unveiled that FH is a protective factor against DM [23], The association between long-term statin therapy and increased risk of developing type 2 DM is controversial. Many studies have reported long term statin therapy results in modest increase in the risk of DM which is time and dose dependent [25, 26]. The presence of additional predisposing factors such as obesity and advanced age increase the risk of statin-mediated hyperglycemia [27]. On the other hand, Fuentes et al. [28] have mentioned that long term statin therapy in FH patients does not increase the risk of developing DM, suggesting that this relationship may vary depending on the underlying metabolic and genetic factors.

The combination of the aforementioned factors could lead to the conclusion that DM may lead to the overestimation of the DLCN score (Positive family history of CAD, high LDL and premature CAD), leading to a false clinical suspicion of FH with a subsequent negative genetic test. Thus, this highlights the importance of targeting young age, positive family history of PCAD and diabetes-free patients in any future studies involving genetic testing in PCAD patients.

The absence of pathogenic and likely pathogenic variants in a proportion of the studied patients in our patients could be attributed to more than one factor; non-genetic PCAD caused by environmental factors, polygenic cause of hypercholesterolemia, which accounts for the majority of cases where DLCN scores suggest probable or definite FH but genetic testing for common genes causing monogenic hypercholesterolemia is negative, variants in other genes related to lipid metabolism and not included in our analysis, or FH caused by rearrangements or variants in promoter regions or introns, which were not evaluated in the current study [29].

Table 8 shows the differences in the prevalence of clinically and genetically diagnosed FH among different populations and shows the proportion of those accurately diagnosed by DLCN scores after genetic confirmation. DLCN diagnosis alone could overestimate the true prevalence of FH. This shows the need for DNA testing confirmation for those cases with probable or definite DLCN scores. This is consistent with our findings that 11 out of 38 patients (28.9%) with a definite/probable DLCN score harbored Pathogenic/Likely pathogenic (Table 5).

**Table 8:** Prevalence of FH in patients with PCAD in different population studies

References	Study location	FH − Genetic testing	FH − DLCN	Genetic + DLCN
Cui et al. [30]	Beijing, China	10/225 (4.4)	12/225 (5.3)	4/12 (33.3)
Amor-Salamanca et al. [14]	Madrid, Spain	9/103 (8.7)	28/103 (27.2)	5/28 (17.8)
Present study	Cairo and Alexandria, Egypt	14/94 (14.89)	38/96 (39.5)	11/38 (28.94)

*FH* Familial Hypercholesterolemia, *DLCN* Dutch Lipid Clinic Network

DLCN positive results were considered to be those with probable or definite classification

Values are presented as n/total (%)

The differences in the percentage of cases diagnosed through genetic testing in these studies can be explained by differences in their patient selection criteria and genetic testing methods. Cui et al. utilized Sanger sequencing for all exons of *LDLR, PCSK9, LDLRAP1* and exon 26 of *APOB* in males ≤ 55 years old and females ≤ 60 years old [30]. Amor-Salamanca et al. performed Next Generation Sequencing for the promoter, exons and exon–intron boundaries for *LDLR, PCSK9, APOB, LDLRAP1, LIPA, STAP1,* and *APOE* in male and female patients aged < 65 years [14]. Accordingly, the comparison between these different studies is not an accurate estimate of the prevalence of genetically confirmed FH in different studies among different populations.

In the current study, the sensitivity, specificity, PPV and NPV for patients with probable/definite FH were 78.6%, 67.14%,32.3% and 94% respectively. Chan et al. conducted a study in 2018 on 885 patients and reported the sensitivity, specificity, PPV and NPV for patients with definite/probable FH to be 91.8%, 43.5%, 41.2% and 92.4% [31]. The current study has lower sensitivity and higher specificity which can be attributed to the smaller sample size especially after the exclusion of the patients with VUS. However, the PPV and NPP in the 2 studies are comparable. Accordingly, DLCN criteria can be a good clinical screening tool but it requires a confirmatory genetic test, as the high sensitivity and NPV can identify most of the true positive cases and exclude the true geneticaly negative cases of FH. However, the moderate specificity and low PPV can lead to many false positive cases. This observation needs further confirmation by increasing the sample size and analysis of the whole genes including the regulatory sequences, introns and large deletions and rearrangement.

Early diagnosis of FH is of utmost importance as it will have a substantial impact on the patients’ prognosis. It allows the patient to take protective measures that will greatly reduce future risk of CAD and its associated complications. In addition, this early diagnosis will allow recognition of inheritance patterns and subsequent inheritance risk calculation among family members with appropriate genetic counseling. It will also allow for cascade screening to take place to detect family members carrying the same variant and offering early and preventive management. In order to reduce premature CAD morbidity and mortality, NICE guidelines advise genetic testing for those with DLCN scores > 5 and cascade genetic testing for first, second and, if possible, third degree relatives [32].

In the present study, 79% of the patients with pathogenic/likely pathogenic variants have baseline LDL > 190 mg/d. In addition, the patient with the ***APOB*** variant has lower LDL levels compared to the patients with ***LDLR****, ****PCSK9*** and ***LDLRAP1*** variants. Other Studies have shown that LDL levels vary among patients harboring different types of variants. One study by Cui et al. showed that carriers of *LDLR* variants had significantly higher LDL levels than those with ***APOB*** variants (5.72 vs 4.93 mmol/L, respectively) and that 60% of genetically-confirmed FH had LDL levels of over 190 mg/dl [30]. Two other studies by Abul-Husn et al. and Khera et al. found that only 45% of genetically confirmed FH cases had LDL levels of over 190 mg/dL [8, 33]. This points towards LDL being insufficient on its own as a sole diagnostic tool for FH.

Globally, ***LDLR*** is the most common mutant gene causing monogenic FH in 80–85% of molecularly diagnosed patients, followed by ***APOB*** gene in almost 5% of the cases and each of the ***PCSK9****, ****LDLRAP1****, ****ABCG5*** and ***ABCG8*** is mutant in less than 1% of molecularly diagnosed patients [34]. In our study ***LDLR*** gene is mutant in 64% (9/14) of patients with pathogenic or likely pathogenic variants, ***LDLRAP1*** is mutant in 21% (3/14), ***PCSK9*** in 7% (1/14) and ***APOB*** gene in 7% (1/14) of the cases. In the current study, six patients had a homozygous variant, 3 in the ***LDLR*** gene (Autosomal dominant FH) and 3 in the ***LDLRAP1*** gene (Autosomal recessive hypercholesterolemia) representing 42.85% (6/14) of patients with pathogenic/likely pathogenic variants which could be attributed to the high prevalence of consanguineous marriage in the current cohort (33.3%), in concordance with the reported prevalence of consanguinity among Egyptians (35.3%) [35].

In the current study, 5 VUS can be considered as hot VUS and warrant further analysis; ***ABCG5*:c**.**1426G > C (p**.**Val476Leu)** is a novel variant that was found in a patient with another VUS in the same gene ***ABCG5*:c**.**1285G > A (p**.**Ala429 Thr)** with a low population frequency. The latter variant was suggestive of pathogenicity by insilico analysis and was reported in an 8-year-old girl with hypercholesterolemia in Poland [36]. However, in the current study there is no confirmation whether the 2 variants are in cis or trans and according we can comment on their role in causing autosomal recessive phenocopy of FH.

Although ***ABCG5/8*** genes cause autosomal recessive sitosterolemia, heterozygous carriers of ***ABCG5/8*** variants may have a small effect on elevating LDL-C levels, resulting in a less severe hypercholesterolemic phenotype [37]. Tada H et el. 2019 reported that patients with homozyous or compound heterozyous rare and delterious variants in ***ABCG5/8*** genes may have a presentation mimicking FH, and that presence of variants in ***ABCG5/8*** genes in addition to known FH genes mutation may increase the severity of the phenotype in FH patients [38].

***LDLR*:c**.**431 C > T (p**.**Pro144Leu)** is a missense variant that was reported as VUS three times in Clinvar and with an allele frequency of 0.000003718 in GnomAD. ***LDLR*:c**.**1546G > A (p**.**Gly516Ser)** is another missense variant in *LDLR* with a gnomAD exome frequency of 0.0000716 (PM2), both variants are present in functional domains and present in variant hotspot. According variants are considered as hot VUS and warrant further analysis through segregation analysis and/or functional studies.

Functional studies were reported for many ***APOB*** variants some of which were detected in the current study, including: ***APOB*:c**.**11477 C > T (p**.**Thr3826Met)** which shows decreased LDL-C binding and internalization which shows incomplete penetrance [39]. ***APOB*:c**.**13477 CAG(1) (p**.**Gln4494 del** shows ~ 50% reduced binding and uptake of LDL(39). While ***APOB*:c**.**2981 C > T(p**. **Pro994Leu)** shows a neutral effect [40].

***APOB*:c**.**9386 C > G (p**.**Pro3129 Arg) and *APOB:*c**.**2062 A > T (p**.**Ile688Phe)** are novel missense variants in the current study. However, other missense variants were reported in the same codons as VUS in Clinvar. The functional studies and segregation analysis may result in reclassification of these novel variants.

In this study, four *APOE* variants were detected; **(*APOE*):c**.**388 T > C (p**.**Cys130 Arg)**, is a common variant that gives rise to isoform epsilon 4 of *APOE* and does not lead to FH, **(*APOE*):c**.**487 C > T (p**.**Arg163 Cys)**, which is usually linked to Familial Dysbetalipoproteinemia, in which LDL levels are usually low. This variant,however, was reported once in one family with autosomal dominant FH [41]. **(*APOE*):c**.**688G > A (p**.**Glu230Lys)** variant which has been previously reported to be linked with autosomal dominant familial combined hyperlipidemia in one family was also detected in a single patient in the present study. (***APOE*):c**.**526 C > T (p**.**Arg176 Cys**) variant is a drug response variant that does not cause FH. Khalil et al. [41] reported p.Leu167 del as a common cause of autosomal dominant FH in patients lacking variants in LDLR, PCSK9, or APOB, however, it was not detected in any of our cohort.

Control of LDL levels is an essential aspect of FH management. LDL reduction serves the purpose of reducing CAD morbidity and mortality. This leads to improved prognosis and reduced rate of complications. NICE guidelines for primary prevention of CAD and for management of FH recommend statins for individuals without FH with a 10% or more 10-year risk of coronary events and high dose statins for any individual diagnosed with FH with the aim of 50% or more reduction in baseline LDL levels [32, 42].

The importance of LDL control is highlighted by the discovery that clinically-diagnosed FH patients with CAD had a 2 times higher risk of CAD recurrence within one year of discharge than those without FH [43]. It was also shown that most FH patients do not achieve desired LDL on-treatment targets. Amor-Salamanca et al. showed that among 9 patients with genetically-confirmed FH, only 1 patient achieved desired LDL levels [14].

Another study by Cui et al. also showed that only 1 patient achieved desirable levels out of 10 FH patients (10%). Their study also included 215 CAD patients who tested negative for FH variants. Of these 215, 76 patients (35.35%) had uncontrolled LDL levels on-treatment [30]. This is concordant with the findings of our study as it was found that none of the 14 patients with pathogenic/likely pathogenic variants achieved desirable LDL levels of < 55 mg/dL and only 6 patients from the entire cohort of 96 patients (6.25%) achieved desirable LDL control. These findings point towards an inadequate management of dyslipidemia in PCAD patients in general, and in FH patients in particular. An extended study is thus warranted to assess the adequacy of lipid management among confirmed FH patients.

Monitoring lipid levels should be recommended to first degree relatives with FH. For those diagnosed and/or on treatment for FH, annual assessment of CAD symptoms, risk factors, fasting lipid levels, treatment adherence, side effects and management plan should be carried out [32].

In Egypt, this is the first study to address the genetic prevalence of FH in premature CAD. The primary clinical study of FH among premature CAD by Reda et al. was based on the electronic data available on a cross-sectional nationwide study called the CardioRisk project. Presence of premature CAD and LDL levels were used to calculate a DLCN score for 2743 patients and it was found that 4 patients had definite FH (0.1%), 7 patients had probable FH (0.25%), 461 patients had possible FH (16.8%) and 1271 patients had unlikely FH (82.85%) [10]. These findings coincide with our study’s findings that most patients with CAD would fall under the diagnosis of unlikely or possible FH (60.02%) when relying only on the DLCN score.

The reclassification of the VUS in our study to likely pathogenic or likely benign would ensure optimal diagnosis and management of FH patients. This can be achieved through functional studies, segregation analysis, in addition to studying the allele frequency in the Egyptian population, which can be achieved through comparison of the results to Egyptian Genome variant frequency anticipated to be available from the Egyptian Genome national project [44].

In addition, hypercholesterolemia polygenic risk score should be applied for all patients with negative or VUS results on genetic testing as 20–30% of patients with clinical diagnosis of FH have polygenic causes of their hypercholesterolemia. [45, 46].

In conclusion, this is the first pilot study in Egypt to conduct genetic testing for FH variants in PCAD patients to date. More extended studies need to be undertaken in different tertiary centers to estimate the prevalence and determine the genetic profiling of FH among Egyptians nationwide and its impact on management of CAD.

### Limitations of the study

The main limitation of the study involved the precise calculation of DLCN scores for all study participants. Issues that arose included limited patients’ knowledge of their family history of tendinous xanthomas, arcus cornealis, and dyslipidemia, therefore DLCN scores presented in this study are likely to be underestimated. Some patients had missing baseline records of their lipid profiles, and so, DLCN was calculated using lipid profiles measured following their participation in this study. Possible rearrangements or variants in promoter regions or introns were not evaluated in the current study which could contribute to a missed genetic diagnosis of FH in the negative cases. Carrier detection in families with genetically-confirmed FH and segregation analysis for patients with VUS were not performed in the current study.

## Conclusions

Based on our findings, the genetic prevalence of FH in our cohort is 14.89%, DLCN clinical scoring system is a good screening tool but requires confirmatory genetic testing for the accurate diagnosis and management of FH and is also essential for identifying high risk cases for the aim of implementing preventive personalized management approach. Optimal inclusion and exclusion criteria for future studies on FH are highlighted. Genetic testing in the study revealed variants of uncertain significance that require further validation for potential pathogenicity. FH is underdiagnosed and undertreated in Egypt and larger studies are required to uncover the true prevalence of genetically confirmed FH in the Egyptian population and to identify the most common pathogenic variants causing FH in Egypt in order to customize a targeted screening panel and personalize the management plan.

## Supplementary Information

Additional file 1

## Data Availability

The datasets used and/or analyzed during the current study are available from the corresponding author upon request.

## Abbreviations

ACMG: American College of Medical Genetics
CAD: Coronary artery disease
DLCN: Dutch lipid clinic network
FH: Familial hypercholestrolemia
MEDPED: Make early diagnosis to prevent early death criteria
PCAD: Premature coronary artery disease
SBR: Simon Broome register
NGS: Next generation sequencing
