# Supplementary material for: Correlation between clinical classification and genetic analysis of familial hypercholesterolemia in premature coronary artery disease in a cohort of Egyptian patients
Source: Hum Genomics. 2025 Jun 14;19:66. doi: 10.1186/s40246-025-00769-y (PMC12167582; doi:10.1186/s40246-025-00769-y)
Supplement: Supplementary file 1 — Additional file 1 [file 40246_2025_769_MOESM1_ESM.docx]

**Supplemental data**

**Supplemental tables**

**Table I: Rare VUS** **detected in the 7 FH genes analysed in the current study**

| **VUS** | | | | | | | | |
| --- | --- | --- | --- | --- | --- | --- | --- | --- |
| **Variant ID** | **Chromosomal location** | **Ref allele** | **Alt allele** | **Mutation type** | **Functional analysis/ Clinvar & ACMG classification** | **Allele frequency in population**  **(GnomAD)** | **Patients** | **Insilico prediction** |
| ***LDLR*** | | | | | | | | |
| NM_000527.5:c.431C>T (p.Pro144Leu) | 19: 11216013 | C | T | SNV  missense | VUS  PM1,PM2, PS4_supporting | 0.000003718 | Heterozygous mutation in 1 patient | REVEL: benign supporting  0.392 |
| NM_000527.5:c.1546G>A  (p.Gly516Ser) | 19: 11224398 | G | A | SNV  missense | Conflicting classifications of pathogenicity  VUS(8);  Likely benign(3);  Benign(1)  PM2,PS4_supporting | 0.00007125 | Heterozygous mutation in 1 patient | REVEL: benign moderate  0.347  MutationTaster: uncertain  SIFT: uncertain |
| ***APOB*** | | | | | | | | |
| NM_000384.3:c.9386C>G  ( p.Pro3129Arg) | 2:21230354 | G | C | SNV  Missense | VUS  PM2, PP3 | Not found | Heterozygous mutation in a patient with probable FH | SIFT: pathogenic MutationTaster: benign |
| NM_000384:c.7190T>C (p.Ile2397Thr) | 2:21232550 | A | G | SNV  missense | VUS  PM2, BP4,BP5 | 0.000006199 | Heterozygous mutation in 1 patient with definite FH and homozygous ***LDLR*** pathogenic mutation | SIFT:  benign supporting  MutationTaster: uncertain |
| NM_000384.3:c.3972G>T (p.Lys1324Asn) | 2:  21236276 | C | A | SNV  missense | Conflicting classifications of pathogenicity  VUS(4); Likely benign(1) (Clinvar)  VUS  PM2,BP4 | 0.00004461 | Heterozygous mutation in 1 patient | Polyphen benign  MutationTaster  Uncertain |
| NM_000384.3:c.10811C>T (p.Ala3604valine) | 2: 21228929 | G | A | SNV  missense | VUS  PM2, BP4, BP5 | 6.196e-7 | Heterozygous  reported in 1 patient with homozygous pathogenic ***LDLR*** | Polyphen: benign |
| APOB:c.12698C>A (p.Ser4233*) | 2: 21225596 | G | T | SNV  Nonsense | VUS  PVS1, PM2 | Not reported | Heterozygous mutation in a patient with possible FH | APOB:c.12698C>A (p.Ser4233*) |
| NM_000384.3:c.9564T>A  (p.Asn3188Ser) | 2: 21230176 | A | T | SNV  missense | VUS  PM2, BP4,BP5 | 0.000001239 | Heterozygous mutation in 1 patient with homozygous pathogenic ***LDLR*** mutation | SIFT:  benign moderate  MutationTaster: uncertain |
| NM_000384.3:c.3671G>A (p.Arg1224Gln) | 2:21237970 | C | T | SNV  missense | VUS  PM2, BP4 | 0.00002292 | Heterozygous mutation in 1 patient with ***APOE*** VUS | Polyphen:  benign |
| NM_000384.3:c.2062A>T  (p.Ile688Phe) | 2:21250705 | T | A | SNV  missense | No data available on Clinvar  PM2, BP4 | Not found | Heterozygous mutation in 1 patient | DANN: benign moderate  SIFT: benign moderate  MutationTaster: benign supporting |
| ***APOE*** | | | | | | | | |
| NM_000041.4:c.688G>A (p.Glu230Lys) | 19: 45412241 | G | A | SNV  missense | VUS  PM2 | 0.00002415 | Heterozygous mutation in 1 patient | Polyphen: benign  SIFT: tolerated |
| ***ABCG5*** | | | | | | | | |
| NM_022436.3:c.1285G>A (p.Ala429Thr) | 2: 44051091 | G | A | SNV  missense | VUS  PM2 | 0.0000397 | Compound Heterozygous mutation in 1 patient with another VUS in ***APOE*** gene | Polyphen  possibly_damaging  SIFT  Deleterious |
| NM_022436.3:c.1426G>C (p.Val476Leu) | 2: 44049973 | G | C | SNV  Missense | VUS  PM2 | Not found |  | SIFT: benign  MutationTaster:  uncertain |
| ***ABCG8*** | | | | | | | | |
| NM_022437.3:c.1226A>G  p.(Asn409Ser) | 2:44100940 | A | G | SNV  missense | Conflicting classification of pathogenicity:VUS(2); Likely benign(1)  PM2, BP6, BP4 | 0.00007435 | Compound heterozygous mutation in 1 patient | AlphaMissense: pathogenic moderate  SIFT: uncertain  MutationTaster: uncertain |
| NM_022437.3:c.1619T>C  p.(Phe540Ser) | 2:44102415 | T | C | SNV  missense | VUS  PM2 | 0.000009913 |  | SIFT: uncertain  MutationTaster: benign supporting |
| ***LDLR***= Low-density lipoprotein receptor; ***ApoB***= Apolipoprotein B; ***ApoE***= Apolipoprotein E; ***ABCG5/8***= ATP binding cassette subfamily G member 5/8; **SNV**= single nucleotide variant; **VUS**= variant of uncertain significance; FH= Familial hypercholesterolemia; **BP**= benign supporting : **BS**= benign strong ; **PM=** pathogenic moderate ; **PP=** pathogenic supporting  Supplemental Table I: Rare Variants of Uncertain significance | | | | | | | | |

**Table II: Common VUS/ rare synonymous and intronic variants detected in the 7 FH genes analysed in the current study**

| **VUS** | | | | | | | | |
| --- | --- | --- | --- | --- | --- | --- | --- | --- |
| **Variant ID** | **Chromosomal location** | **Ref allele** | **Alt allele** | **Mutation type** | **Clinvar & ACMG classification** | **Allele frequency in population**  **(GnomAD)** | **Patients** | **Insilico prediction** |
| ***LDLR*** | | | | | | | | |
| NM_000527.5:c.2390-34dup | 19:11240150 | C | CG | Insertion in a non-coding region | No data available on Clinvar  PM2, BP7 | Not found | Heterozygous mutation in 1 patient | No data available |
| ***APOB*** | | | | | | | | |
| NM_000384.3:c.11477C>T  (p.Thr3826Met) | 2: 21228263 | G | A | SNV  missense | Conflicting classifications of pathogenicity  VUS(3); Benign(3); Likely benign(8) (Clinvar)  ACMG classification:  Benign  BP6,BS1,BS2  Functional studies: decreased LDL binding and internalization | 0.002406 | Heterozygous  mutation in 1 patient with homozygous pathogenic ***LDLRAP1*** mutation | Polyphen: probably damaging  Revel: benign |
| NM_000384.3:c.2981C>T  (p. Pro994Leu) | 2:21242613 | G | A | SNV  missense | Conflicting classifications of pathogenicity  VUS(7); Likely benign(6); Benign(2)  PM2, BP6, BP4  Functional studies: neutral effect | 0.001014 | Heterozygous mutation in 1 patient | SIFT: pathogenic supporting  MutationTaster: uncertain  REVEL: benign moderate |
| NM_000384.3:c.3279C>T  (p.Thr1093=) | 2:21239364 | G | A | SNV  Synonymous | No data available on Clinvar  PM2, BP7, BP4 | Not found | Heterozygous mutation in 2 patients with another VUS in ***APOE*** | DANN: benign moderate  CADD: benign strong |
| NM_000384.3:c.948A>G  (p.Thr316=) | 2:21256347 | T | C | SNV  Synonymous | No data available on Clinvar  PM2, BP7, BP4 | 6.195e-7 | Heterozygous mutation in 1 patient | DANN: benign strong  CADD: benign strong |
| ***LDLRAP1*** | | | | | | | | |
| NM_015627.3:c.605C>A (p.Ser202Tyr) | 1: 25889633 | C | A | SNV  missense | Conflicting classifications of pathogenicity  Likely pathogenic(1); VUS(2); Benign(1); Likely benign(1) (Clinvar)  Benign  PM5,BP4,,BS1, | 0.0008389 | Homozygous mutation in 1 patient | MutationTaster: benign moderate  SIFT: uncertain |
| ***APOE*** | | | | | | | | |
| NM_000041.4:c.487C>T (p.Arg163Cys) | 19:45412040 | C | T | SNV  missense | Conflicting classifications of pathogenicity  VUS(1); Benign(5); Likely benign(1)  ACMG Classification  VUS  BA1, BS2, BP6, PM5, PP3, PP5 | 0.001245 | Heterozygous mutation in 2 patients, one of them has another VUS in ***APOB*** | Polyphen: probably damaging  SIFT: deleterious |
| NM_000041.4:c.388T>C (p.Cys130Arg) | 19: 45411941 | T | C | SNV  missense | Conflicting classifications of pathogenicity;  Pathogenic(1); Likely pathogenic(2); VUS(2  ACMG Classification:  VUS  BA1, PP5 | 0.1485 | Heterozygous in 14 patients, two of them have heterozygous VUS in the ***APOB*** gene, one has compound heterozygous variants in ***ABCG5*** and one has pathogenic heterozygous ***LDLR*** mutation. | SIFT, MutationTaster  benign |
| NM_000041.4:c.526C>T  (p.Arg176Cys) | 19:45412079 | C | T | SNV  missense | Drug response for atorvastatin  BP6, BP4 | 0.07424 | Heterozygous mutation in 9 patients and homozygous mutation in 1 patient | MutatonTaster: benign moderate  SIFT: uncertain |
| ***LDLR***= Low-density lipoprotein receptor; ***ApoB***= Apolipoprotein B; ***ApoE***= Apolipoprotein E; ***LDLRAP1*** = Low-density lipoprotein adaptor protein 1; **SNV**= single nucleotide variant; **VUS**= variant of uncertain significance; FH= Familial hypercholesterolemia; **BA**=Benign Standalone ; **BP**= benign supporting : **BS**= benign strong ; **PM=** pathogenic moderate ; **PP=** pathogenic supporting  Supplemental Table II: Common variants of Uncertain significance | | | | | | | | |

**Table III: Heterozygous VUS in autosomal recessive FH genes**

| **VUS** | | | | | | | | |
| --- | --- | --- | --- | --- | --- | --- | --- | --- |
| **Variant ID** | **Chromosomal location** | **Ref allele** | **Alt allele** | **Mutation type** | **Clinvar & ACMG classification** | **Allele frequency in population**  **(GnomAD)** | **Patients** | **Insilico prediction** |
| ***ABCG5*** | | | | | | | | |
| NM_022436.3:c.235G>A (p.Gly79Arg) | 2:44065003 | C | T | SNV  missense | VUS  PM2 | 0.0005725 | Heterozygous mutation in 1 patient | Polyphen probably_damaging  SIFT deleterious |
| NM_022436.3:c.1411G>A (p.Val471Ile) | 2: 44049988 | C | T | SNV  missense | Conflicting classifications of pathogenicity: VUS(2); Likely benign(2)  ACMG: VUS  BP4,PM2 | 0.0002980 | Heterozygous mutation in 1 patient with ***APOE*** VUS | SIFT: benign  MutationTaster: uncertain |
| NM_022436.3:c.392A>G (p.Tyr131Cys) | 2: 44059096 | T | C | SNV  missense | VUS  PM2, PP3, BP5 | 0.00004581 | Heterozygous mutation in 1 patient with homozygous pathogenic ***LDLRAP1*** mutation | Polyphen2  Deleterious  Revel  Deleterious  SIFT  Uncertain |
| NM_022436.3: c.1486G>T (p.Val496Phe) | 2:  44047217 | C | A | SNV  Missense | VUS  PM2 | Not found | Heterozygous mutation in a patient with probable FH | SIFT: benign MutationTaster: uncertain |
| ***ABCG8*** | | | | | | | | |
| NM_022437.3:c.1648G>A (p.Ala550Thr) | 2:44102444 | G | A | SNV  missense | VUS  PM2 | 0.0001636 | Heterozygous mutation in 1 patient | Polyphen possibly damaging  SIFT: tolerated |
| NM_022437.3:c.613G>A  p.(Val205Met) | 2:44079544 | G | A | SNV  missense | VUS  PM2, PP3 | 0.00003039 | Heterozygous mutation in 1 patient with another VUS in ***APOE*** | SIFT: uncertain  SIFT4G: pathogenic supporting  MutationTaster: uncertain |
| NM_022437.3:c.1315G>A  p.(Gly439Arg) | 2:44101029 | G | A | SNV  missense | No data available on Clinvar  PM2 | 6.195e-7 | Heterozygous mutation in 1 patient with another VUS in ***APOE*** | SIFT: uncertain  MutationTaster: benign supporting |
| NM_022437.3:c.515A>C  p.(Gln172Pro) | 2:44078915 | A | C | SNV  missense | No data available on Clinvar  PM2 | Not found | Heterozygous mutation in 1 patient with another VUS in ***APOE*** | MutationTaster: benign moderate  SIFT: benign moderate |
| ***ABCG5/8***= ATP binding cassette subfamily G member 5/8; **SNV**= single nucleotide variant; **VUS**= variant of uncertain significance; FH= Familial hypercholesterolemia; **BA**=Benign Standalone ; **BP**= benign supporting : **BS**= benign strong ; **PM=** pathogenic moderate ; **PP=** pathogenic supporting  Supplemental Table III: heterozygous variants of uncertain significance in autosomal recessive FH genes | | | | | | | | |

| **Family history:** | |
| --- | --- |
| First degree relative with LDL >95th percentile for age and gender | 1 |
| First degree relative with premature CAD (men<55 years, women <60 years) | 1 |
| First degree relative with tendon xanthomas and/or arcus cornealis  And/or Children <18 years old with LDL >95th percentile | 2 |
| **Clinical history:** | |
| Patients with premature coronary artery disease | 2 |
| Patients with premature cerebral or peripheral vascular disease | 1 |
| **Physical examination:** | |
| Tendon xanthomas | 6 |
| Arcus cornealis before age <45-year-old | 4 |
| **LDL-C level in mg/dl** | |
| >330 | 8 |
| ~250-329 | 5 |
| ~190-249 | 3 |
| ~155-189 | 1 |
| **DNA analysis** | |
| Causative mutation in the LDLR | 8 |
| <3 points: no diagnosis, 3-5 points: possible FH, 6-8 points: probable FH, >8 points definite FH | |

Supplemental Table IV: DLCN diagnostic criteria for FH. ^48^
